# Structure of Microbial Communities and Biological Activity in Tundra Soils of the Euro-Arctic Region (Rybachy Peninsula, Russia)

**DOI:** 10.3390/microorganisms11051352

**Published:** 2023-05-22

**Authors:** Maria V. Korneykova, Vladimir A. Myazin, Nadezhda V. Fokina, Alexandra A. Chaporgina, Dmitry A. Nikitin, Andrey V. Dolgikh

**Affiliations:** 1 Smart Urban Nature Research Center, RUDN University, 6 Miklukho-Maklaya St, Moscow 117198, Russia; korneykova.maria@mail.ru; 2 Department of Ecology of Microorganisms, Institute of North Industrial Ecology Problems–Subdivision of the Federal Research Centre “Kola Science Centre of Russian Academy of Science”, Apatity 184209, Russia; myazinv@mail.ru (V.A.M.); nadezdavf@yandex.ru (N.V.F.); sashka-26.11.91@mail.ru (A.A.C.); 3 Department of Soil Biology and Biochemistry, V.V. Dokuchaev Soil Science Institute, Moscow 119017, Russia; dimnik90@mail.ru; 4 Department of Soil Geography and Evolution, Institute of Geography, Russian Academy of Sciences, Moscow 119017, Russia

**Keywords:** extreme ecosystems, microbial biomass, number of culturable bacteria and fungi, β-glucosidase, dehydrogenase and urease activity in soil

## Abstract

The relevance of the Arctic regions’ study is rapidly increasing due to the sensitive response of fragile ecosystems to climate change and anthropogenic pressure. The microbiome is an important component that determines the soils’ functioning and an indicator of changes occurring in ecosystems. Rybachy Peninsula is the northernmost part of the continental European Russia and is almost completely surrounded by Barents Sea water. For the first time, the microbial communities of the Entic Podzol, Albic Podzol, Rheic Histosol and Folic Histosol as well as anthropogenically disturbed soils (chemical pollution and human impact, growing crops) on the Rybachy Peninsula were characterized using plating and fluorescence microscopy methods, in parallel with the enzymatic activity of soils. The amount and structure of soil microbial biomass, such as the total biomass of fungi and prokaryote, the length and diameter of fungal and actinomycete mycelium, the proportion of spores and mycelium in the fungal biomass, the number of spores and prokaryotic cells, the proportion of small and large fungal spores and their morphology were determined. In the soils of the peninsula, the fungal biomass varied from 0.121 to 0.669 mg/g soil. The biomass of prokaryotes in soils ranged from 9.22 to 55.45 μg/g of soil. Fungi predominated, the proportion of which in the total microbial biomass varied from 78.5 to 97.7%. The number of culturable microfungi ranged from 0.53 to 13.93 × 10^3^ CFU/g in the topsoil horizons, with a maximum in Entic Podzol and Albic Podzol soils and a minimum in anthropogenically disturbed soil. The number of culturable copiotrophic bacteria varied from 41.8 × 10^3^ cells/g in a cryogenic spot to 5551.3 × 10^3^ cells /g in anthropogenically disturbed soils. The number of culturable oligotrophic bacteria ranged from 77.9 to 12,059.6 × 10^3^ cells/g. Changes in natural soils because of anthropogenic impact and a change in vegetation types have led to a change in the structure of the community of soil microorganisms. Investigated tundra soils had high enzymatic activity in native and anthropogenic conditions. The β-glucosidase and urease activity were comparable or even higher than in the soils of more southern natural zone, and the activity of dehydrogenase was 2–5 times lower. Thus, despite the subarctic climatic conditions, local soils have a significant biological activity upon which the productivity of ecosystems largely depends. The soils of the Rybachy Peninsula have a powerful enzyme pool due to the high adaptive potential of soil microorganisms to the extreme conditions of the Arctic, which allows them to perform their functions even under conditions of anthropogenic interference.

## 1. Introduction

The study of the Polar Regions’ soil microbiome is one of the most current areas of modern microbiology [1]. Due to extreme conditions, microorganisms are the leading force in the cycle of biogenic elements of ecosystems there [2,3]. The prokaryote and microfungal taxonomic diversity of the northern territories of America, Canada, Finland, Norway as well as Antarctica is relatively well studied [3,4,5]. At the same time, the quantitative indicators of the soil microbial community of the Arctic territories (number of microorganisms, amount and structure of microbial biomass, level of basal respiration, etc.), which play an important role in the functioning of ecosystems, have not been practically studied [2,6,7]. For example, microbial biomass is an important parameter of the concentration of macronutrients cycle in the soil [7] and makes it possible to assess the level of ecosystem productivity [8]. It should be noted that Arctic soils are characterized by a low abundance and diversity of microbial communities, so their study reveals the simplest ecological patterns [9].

Plating methods on nutrient media have advantages and disadvantages. It is known that these methods can isolate only a small part of the microbial community, but at the same time, they make it possible to characterize the community structure using synecological characteristics, to isolate pure cultures and to characterize their role in the soil processes [10]. In addition, bacteria capable of growing on nutrient media, which includes many aerobic organotrophic and fast-growing R-strategists, are the most reactive component of bacterial community and are able to quickly respond to environmental changes [11]. Their reaction can be expressed both in a change in the community structure, including a change in the proportion of ecological and trophic groups, and in the magnitude of short-term fluctuations in abundance [12]. Moreover, with an increase in the level of environmental pollution, the proportion of cultured forms of bacteria may increase [13].

The Rybachy Peninsula is the extreme northwestern site of continental European Russia [14]. The vegetation cover of the peninsula is tundra in the territory of eastern Fennoscandia, occupying a relatively narrow strip along the Barents Sea [15]. The soil cover of the Rybachy Peninsula has been studied in sufficient detail; however, its biological activity has practically not been studied [16,17].

Low temperatures and the predominance of moss–lichen associations in the vegetation cover of tundra soils create specific econiches for microorganisms [18] that perform important biogeochemical functions. Their participation in soil formation in the Rybachy Peninsula (including in the formation of rather rare dry peat soils) has been insufficiently studied [16]. The role of fungi and bacteria in the destruction and creation of soil organic matter under these conditions has been overlooked [17]. The biological activity of soils on the Rybachy Peninsula and the role of microorganisms in carbon and nitrogen cycles in these terrestrial ecosystems remain largely unexplored.

This work aimed to study the structure of microbial biomass (assessment of the fungal and prokaryote biomass, the length and diameter of fungal and actinomycete mycelium, the proportion of spores and mycelium in the biomass, the number of fungal spores and prokaryotic cells, the proportion of small and large fungal propagules, as well as the morphology of fungal spores) and number of culturable bacteria and microfungi as well as activity of β-glucosidase, urease and dehydrogenase in anthropogenically modified and background soils of the Rybachy Peninsula (Russia, Murmansk region).

## 2. Materials and Methods

### 2.1. Characteristics of the Climate and Soils

**Figure 1:**
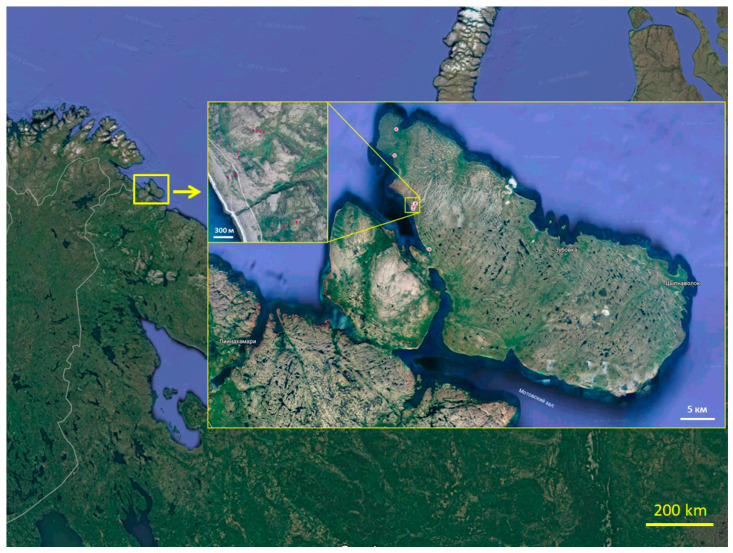
Map of the Rybachy Peninsula (Map data: Google, Maxar Technologies). Note: red dots are sampling sites.

Research was carried out in the Rybachy Peninsula (69°44′ N, 32°30′ E) (Figure 1). The peninsula is composed of Proterozoic sedimentary rocks (shale, quartzite, conglomerate, sandstone).

Plateau uplands are the characteristic forms of the peninsula’s relief. The Rybachy Peninsula belongs to an area with a maritime humid climate formed under the influence of the Barents Sea. The average temperature in July does not exceed +9 °C; in January, it does not fall below −6 °C. Relative air humidity is more than 80%. Average annual rainfall is about 450 mm. Climate of the Rybachy Peninsula is subarctic (cold, no dry season, cold summer—Dfc in Köppen–Geiger classes) [19]. As a result, the area is not underlined by permafrost, but cryogenic forms of microrelief (pattern ground) are often found on the tops of the uplands. The vegetation of the tundra is dominated by shrub, dwarf birch, and lichen varieties. In the relief depressions and valleys, an increase in the proportion of birch thickets is noted.

The seaside climate, conditions of hindered internal drainage and the presence of specific lithogenic factors (soil-forming rocks rich in chemical composition) contributed to the dry peat accumulation and the formation of dry peat soils (Folic Histosol) on the Rybachy Peninsula. In the soil cover of the Rybachy Peninsula tundra, Folic Histosols, Albic and Entic Podzols cover the extensive areas.

The lowered elements of the peninsula’s relief are occupied by hydromorphic peat bog lowland soils (Dystric Rheic Histosols), and peat eutrophic soils (Eutric Rheic Histosols) are formed under the conditions of flowing depressions. A strip of Folic Leptosols of coastal meadow formations is distinguished along the sea, which is associated with the influence of the sea—the transfer of organic material during storms. On the tops of the plateaus, under conditions of blown relief forms, rounded-spotted cryogenic complexes with underdeveloped spot soils (Skeletic Leptic Entic Podzols) are formed.

### 2.2. Sampling

On the territory of the Rybachy Peninsula, 10 sites were selected (Table 1). The choice of the site was determined by the presence of specific natural conditions, which determined the formation of particular types of soils (Folic Histosol, Rheic Histosol, Albic Podzol, Entic Podzol; soil pits RY01–RY10 and RY14) and the level of anthropogenic disturbance (Folic Histosol; soil pits RY11, RY13). Soil pits were excavated at each of the sites for chemical and microbiological analysis and classification according to to the World Reference Base for Soil Resources (WRB) [20]. 

For chemical analysis and the determination of enzymatic activity, soil samples were collected from several soil horizons, brought to the laboratory, dried at 22 °C, and sieved (mesh 2 mm). For microbiological analysis, samples were taken from the same horizons according to the standard sampling procedure [21]. Microbial biomass was determined in the topsoil horizon. Culturable microorganisms and soil enzymatic activity were assessed in the topsoil and subsoil horizons. Soil samples were taken in five replicates.

### 2.3. Prokaryotic Biomass

Number of prokaryotes was counted using the fluorescence method (acridine orange dye) under a Biomed 5 PR LYUM microscope (Moscow, Russia) at a magnification of 1000× (immersion oil) [17]. Excitation of acridine orange complex with prokaryotic RNA occurred at a light wavelength of 490 nm. The wavelengths range of acridine orange–prokaryotic RNA complex emissions was from 500 to 700 nm, and the optimum was from 528 to 531 nm. Only flow prokaryotic cells that were in front of soil particles were considered. For soil particles, it was possible to consider only the multicellular mycelium of actinomycetes, which exceeded size of structural units in soil. Prokaryotic cells were counted in preparations with a soil suspension dilution of 1:100. Prokaryotic cells were desorbed from soil particles by ultrasound at the UZDN-1 installation, where the following parameters were set–2 min, current strength 0.40 A, frequency 22 kHz. For each soil sample, 3 preparations were studied, in each of which 90 microscopic fields of view were analyzed. All fields of view were viewed manually without the use of video cameras and specialized computer programs. This is necessary for better visualization of prokaryotic cells. The number of prokaryotic cells per gram of soil was calculated using Formula (1):

N = S_1_ × a × n/V × S_2_ × C, (1)

where N is the number of prokaryotic cells in a gram of soil; S_1_ is the area of the test preparation on the glass (µm^2^); a is the number of cells for each field of view (the average value for all preparations on the glass was taken); n is the index of dilution of prokaryotic cells (mL); V is the volume of a drop of soil suspension on the preparation (mL); S_2_ is the area of the field of view of the microscope (µm^2^); C is the weight of soil sample in grams.

Number of prokaryotic cells was converted into biomass, considering that each prokaryotic cell has a volume of 0.1 μm^3^ and a mass of 2 × 10^−14^ g [22].

The length of the actinomycete mycelium in 1 g of the sample (LMA) was determined using Formula (2):

M = 4an × 10^10^/p, (2)

where M is the length of the mycelium in 1 g of soil (m/g); a is the average length of mycelium in the field of view; p is the field of view area (µm^2^); n is the dilution index.

To detect bacterial nanoforms (cells ranging in size from 30 to 200 nm), a technique was used that included the isolation of a bacterial complex from a soil suspension, followed by filtration of samples using nuclear membrane filters with a pore size of 200 nm and thickening of the resulting filtrate centrifugation.

### 2.4. Fungal Biomass

Number of spores and length of the fungal mycelium were determined using fluorescence (white calcofluor dye) [23] and a Biomed 5 PR LYUM microscope (Russia) at a magnification of 400×. Excitation of the white calcofluor–fungal chitin complex occurred at a light wavelength of 470 nm. The range of radiation wavelengths of white calcofluor complex with fungal chitin ranged from 540 to 600 nm, and the optimum ranged from 568 to 571 nm. Only those unicellular fungal propagules (spores and yeasts) that were found in front of soil particles and provided a bright glow were taken into account. Behind soil particles, it was possible to detect only multicellular fungal mycelium, which exceeded size of structural units of soil. Fungi cells were counted in preparations with a soil suspension dilution of 1:100. Desorption of fungal cells from soil particles was carried out with an MSV-3500 vortex mixer (Latvia) at a speed of 3500 rpm for 10 min. For each soil sample, 3 preparations were studied, in each of which 90 microscopic fields of view were analyzed. All fields of view were viewed manually without the use of video cameras and specialized computer programs. This is necessary for better visualization of mycelium and fungal spores. The calculation of the number of fungal spores per gram of substrate was carried out according to Formula (3):

M = ((4 × a × n)/p) × 10^10^, (3)

where M is the number of fungal spores per gram of soil; a is the average number of fungal spores in one field of view of the microscope; p is the area of the field of view of the microscope (µm^2^); n is the dilution factor.

Fungal biomass in the analyzed soils was determined with the assumption that the density of fungal spores is 0.837 g/cm^3^ and the specific gravity of fungal mycelium is 0.628 g/cm^3^ [23].

The length of fungal mycelium per gram of soil (LMA) was determined according to Formula (4):

LMA = S_1_ × a × n/v × S_2_ × c × 10^6^, (4)

where S_1_ is the area of the preparation on the glass (µm^2^); a is the average length of the mycelium of fungi in the field of view of the microscope (μm); n is the dilution index of the soil suspension (mL); v is the volume of a drop of soil suspension applied to the preparation glass (mL); S_2_ is the area of the field of view of the microscope (µm^2^); c is the mass of the soil sample (g).

### 2.5. Number of Culturable Microorganisms

The number of colony-forming units (CFU) of culturable microfungi was determined using the plating method on Czapek’s medium with the addition of lactic acid (4 mL/L) to inhibit bacterial growth [22]. For microfungi, a 100-fold dilution of the suspension was used. The number of copiotrophic bacteria was determined using meat peptone agar (MPA), and the number of oligotrophic bacteria on slightly mineralized Aristovskaya medium (AM) [22] For bacteria, a 1000-fold dilution of the suspension was used. Incubation was carried out in thermostats at a temperature of +27 °C for 7–10 days. To isolate psychrotolerant microorganisms, incubation was carried out at +5 °C for 5–6 weeks.

### 2.6. Enzymatic Activity of Soils

To assess the biological activity of soils, two hydrolytic (β-glucosidase, urease) and one oxidoreductase (dehydrogenase) enzymes were determined. The β-glucosidase activity in soil was determined calorimetrically using a method—based on the ability of glucose and fructose, formed as a result of sucrose hydrolysis—of reducing CuO to Cu_2_O. A sample of soil (0.5–2 g, depending on the content of organic matter in the soil) was placed in a 100 mL flask, 20 mL of a buffer solution (pH = 5) and 20 mL of a 2% sucrose solution were added. Three drops of toluene were added to the flask, closed with rubber stoppers, and incubated in a thermostat at 37 °C for 24 h. After that, a 1 mL extract was taken and placed in a 50 mL flask. The volume was brought up to 5 mL with distilled water and 4 mL of copper reagent was added and placed in a water bath for 25 min. After cooling to room temperature, 2 mL of Na_2_HPO_4_ solution and 5 mL of molybdenum solution were added successively. After thorough stirring to remove all carbon dioxide, the sample was left for 1 h to develop maximum color and measure the optical density using a cuvette 5 mm wide at a wavelength of 590 nm [24,25]. Urease activity was determined calorimetrically using Nessler’s reagent. A sample of soil (0.5–2 g, depending on the content of organic matter in the soil) was placed in a 100 mL flask and 20 mL of 2% urea solution in a buffer solution (pH = 7) was added. Three drops of toluene were added to the flask, closed with rubber stoppers, and incubated in a thermostat at 30 °C for 24 h. After that, 80 mL of 2% KCl solution was added and left for 15 min. The extract (0.5–5 mL, depending on the content of organic matter in the soil) was placed in a 50 mL flask. A large amount of distilled water, 2 mL of Rochelle’s salt and 0.5 mL of Nessler’s reagent were added. The solution was calorimetrically analyzed in a cuvette 30 mm wide, at a wavelength of 590 nm [26,27]. Dehydrogenase activity was determined using a colorimetric method based on the reduction of the colorless salt of 2,3,5-triphenyltetrazolium chloride to red triphenylformazan. A sample of soil is placed in a test tube with a volume of 25 mL. Quantities of 10 mg CaCO_3_, 1 mL of 1% glucose solution and 1 mL of 1% solution of 2,3,5-triphenyltetrazolium chloride were added. The determination was carried out under anaerobic conditions using an anaerostat at 37 °C for 24 h. After incubation, 23 mL of ethyl alcohol was added and shaken for 5 min. The solution was filtered and calorimetrically analyzed in a cuvette 5 mm wide, at a wavelength of 540 nm [28].

Preliminarily, for the determination of each enzyme, calibration curves were built using standard solutions: glucose and fructose for β-glucosidase, ammonium for urease, and triphenylformazan for dehydrogenase.

### 2.7. Soil pH and Total Organic Carbon (TOC)

The pH value (soil–water = 1:5) was measured using an electrometric technique (pH/ION Analyser Radelkis OP-300) [24]. The total organic carbon content was determined by ashing the soil with a chromium mixture when heated to 150 °C. The amount of ashed carbon of organic compounds was calculated based on the amount of Cr3+ ions formed as a result of the reaction, which were determined calorimetrically (λ = 590 nm) [29].

### 2.8. Statistical Analysis

The software packages R 4.0.3 (R Core Team, Vienna, Austria) and Microsoft Office Excel were used for statistical analysis and data visualization. Student’s *t*-test with unequal sample variances and Welch’s two-sample t-test with equal sample variances were used to assess the significance of differences in quantitative indicators between the study areas (independent groups). Student’s test was used to compare samples with unequal variances, namely Welch’s test, if there are no differences between the variances of two samples (equality of variances) [30]. The dispersions were checked using Fisher’s test, and the correlation between the level of enzymatic activity, total organic matter content and soil pH were checked using Pearson’s test.

## 3. Results and Discussion

### 3.1. Soil pH and TOC Content

**Figure 2:**
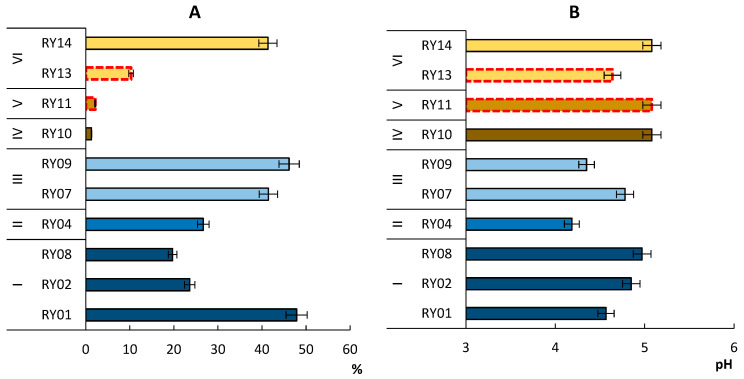
Total organic carbon content (**A**) and pH (**B**) in the soils on the Rybachy Peninsula. Skeletic Folic Leptic Entic Podzol (I), Rheic Leptic Rockic Histosol (II), Skeletic Folic Leptic Albic Podzol (III), Skeletic Leptic Entic Podzol—cryogenic spot (IV), Leptic Hemic Folic Histosol (Technic) (V), Leptic Hemic Folic Histosol (VI). The red dotted line indicates anthropogenically modified areas.

The amount of TOC in the Entic Podzol and Albic Podzol was quite large and reached 47.86% by weight, in the Rheic Histosol—26.67% by weight (Figure 2). Such differences in the content of organic matter in tundra were caused by the differences in type of phytocenoses and the degree of humification of litter. At the same time, in areas under meadow vegetation, the content of TOC in Folic Histosol (RY11) was lower and did not exceed 2.14%. 

The pH value of the topsoil horizons of tundra soils ranged from 4.19 to 5.08, which characterized them as acidic. For Rheic Histosol soil, the minimum pH value was 4.19 (Figure 2). In general, the Entic Podzol and Albic Podzol soils under the moss–lichen and shrub associations were characterized by lower pH than the soils under the birches. Higher pH values were also noted in the soil under a tall grass meadow on the territory of an abandoned settlement and cryogenic spot.

### 3.2. Fungal Biomass

**Figure 3:**
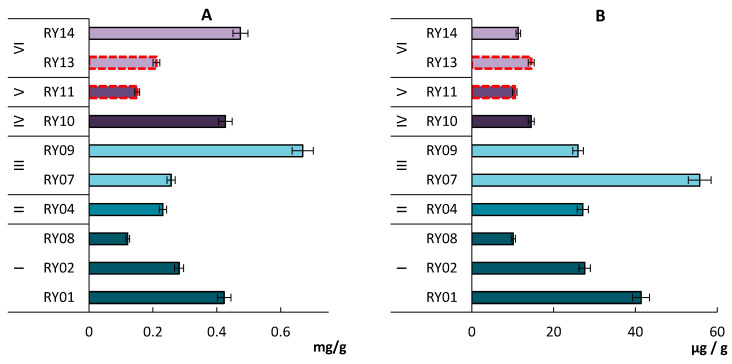
Biomass of fungi (**A**) and prokaryotes (**B**) in topsoil of Skeletic Folic Leptic Entic Podzol (I), Rheic Leptic Rockic Histosol (II), Skeletic Folic Leptic Albic Podzol (III), Skeletic Leptic Entic Podzol—cryogenic spot (IV), Leptic Hemic Folic Histosol (Technic) (V), Leptic Hemic Folic Histosol (VI). The red dotted line indicates anthropogenically modified areas.

In the soils of the Rybachy Peninsula, the fungal biomass changed from tens to hundreds mg/g soil (Figure 3A, Table S1). It was approximately equal to that on the coast of the Barents Sea [31], but less than in the background landscapes of the Kola Peninsula [32,33]. The minimum biomass (0.151 mg/g soil) was found in anthropogenically disturbed soil, and the maximum (0.463 mg/g soil) was found in Albic Podzol. For most samples (67%), the fungal biomass was lower than 0.282 mg/g soil.

The proportion of mycelium in the total fungal biomass changed greatly depending on the type of biotope. It is minimal—14–15%—in terms of fungal biomass in soils of cryogenic spot (RY10) and Entic Podzol (RY08), and maximum—68–78%—in terms of fungal biomass in Entic Podzol (RY01) and Albic Podzol (RY09). The length of fungal mycelium in the soils also varied greatly. It was comparable to that in the soils of the north of Novaya Zemlya [33], but less than on the coast of the Barents Sea [31]. Its lowest values were 14 and 29 m/g of soil in soils of cryogenic spot (RY10), and the highest, 413 m/g of soil, was found in Albic Podzol (RY09) (Table S1). We assume that the TOC content, pH values and type of vegetation cover can primarily explain such a difference in the length of the fungal mycelium for different types of biotopes.

The length of fungal mycelium, as well as the biomass, was minimum in Entic Podzol (RY-08) and Folic Histosol (Technic) (RY-11); however, it was maximum in Albic Podzol (RY-09) and Folic Histosol (RY-14). The portion of thin (less than 3 µm in diameter) fungal mycelium in the soils was relatively large (up to 32%; in some samples, it reached 50–60%) compared to the percentage of that in the soils of Arctic cities located nearby, namely Apatity [34] and Murmansk [31]. A similar trend was found on the Barents Sea coast: mycelium averaged 42% of the total biomass [31]. The reason for this, perhaps, is the urban “heat island”, which is characteristic of cities beyond the Arctic Circle. Extra warmth from a city can stimulate the growth of soil fungi. The absence of basidial buckle mycelium in the soil of the Rybachy Peninsula may indirectly indicate a low number of mycorrhizal symbioses [35].

In this study, we intentionally estimated the size and shape of fungi propagules. This information allows for knowledge of the details of mycobiota ecology. For example, the abundance of small spores may indirectly indicate the stressful state of the community, while the large number of large spores indicates the flourishing of fungi in local conditions [36]. It is also important to be able to distinguish between dormant propagules (spores) and active ones (yeast cells), since, in this way, one can indirectly judge the ratio of living and dead fungi. The number of unicellular fungal propagules (spores and yeasts) in the subaquatic soils was (2–4) × 10^5^ cells/g soil. Most of the fungal propagules were represented by forms of small size (2–3 microns), the proportion of which increased from the topsoil horizons (88–93%) to the subsoil horizons (up to 100%). Large propagules with a diameter of 5–7 µm were found exclusively in topsoil horizons, and their numbers did not exceed 2–11 × 10^4^ cells/g of soil. In the subsoil horizons, most of the fungi were represented by unicellular propagules (spores and yeasts). The absence of large fungal propagules in the deep layers of the soil may be due to the specifics of its structure. With increasing depth, the soil is denser and contains fewer pores, which prevents large spores from penetrating into the lower soil horizons. About 74% of the spores were rounded with a smooth surface; 14% were round and rough; 8% were oval with a smooth surface; 4% had an oval shape with irregularities. No mass budding of yeast was found. In some samples (especially in the moss–lichen hummocky tundra), a large number of hyphae of basidiomycetes are visualized, the specific feature of the mycelium of which is the presence of buckles.

### 3.3. Prokaryote Biomass

The prokaryote biomass in the soils of the Rybachy Peninsula was low, and in other Arctic areas—Taimyr [37], Novaya Zemlya [38] and Franz Josef Land [39]—it ranged from 10.13 to 55.74 μg/g soil (Figure 3B, Table S1). The smallest values of the prokaryote biomass were found in the anthropogenically disturbed soil (RY11). The highest prokaryote biomass was revealed in Albic Podzol (RY07). Similar results were obtained for the podzols of central Russia [40,41]. The average values of prokaryotic biomass in the studied plots differed significantly, which can probably be explained by the content of organic carbon in the soil, as noted in the work of other authors [21]. The biomass of fungi and prokaryotes positively correlates with the content of total organic carbon (r = 0.63; t = 2.31; df = 8, p = 0.05).

In most samples, unicellular forms of prokaryotes dominated (from 78.0 to 99.5%). However, in anthropogenically disturbed (RY11) and cryogenic (RY10) soils, the proportion of actinomycete mycelium reached high values (17.2 and 10.6 %, respectively). The length of the actinomycete mycelium in most samples was low, but this group of microorganisms accounts for most of the number in polar ecosystems [42,43], ranging from 1.47 to 66.32 m/g of soil. The mycelium length of actinomycetes was shorter than in the soils of the cities Murmansk [31] and Apatity [34]. Most prokaryotic cells (up to 76%) were represented by small nanoforms, which is typical for polar ecosystems [44,45].

### 3.4. Total Microbial Biomass

**Figure 4:**
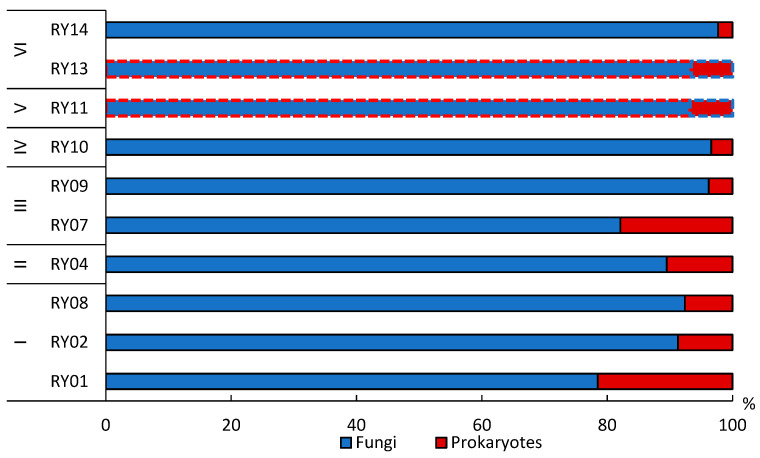
The proportion of fungi and prokaryotes based on biomass of Rybachy Peninsula soils. Skeletic Folic Leptic Entic Podzol (I), Rheic Leptic Rockic Histosol (II), Skeletic Folic Leptic Albic Podzol (III), Skeletic Leptic Entic Podzol—cryogenic spot (IV), Leptic Hemic Folic Histosol (Technic) (V), Leptic Hemic Folic Histosol (VI). The red dotted line indicates anthropogenically modified areas.

The total microbial biomass ranged from 0.131 to 0.695 mg/g soil. The lowest values were found in Entic Podzol (RY-08) and Folic Histosol (Technic) (RY-11) and the highest–in Albic Podzol (RY-09) and in Folic Histosol (RY-14). Fungi were dominant, the proportion of which in the total microbial biomass varied from 78.5 to 97.7% (Figure 4). The lowest microbial biomass was recorded in anthropogenically disturbed soils (Dystric Leptic Hemic Folic Histosol (Technic) and in cryogenic spot soil (Skeletic Leptic Entic Podzol (Arenic)) with poor vegetation cover. The highest values of these parameters were found in the topsoil horizons of Albic Podzols and Entic Podzols.

The active growth of mycelium microorganisms (actinomycetes and filamentous fungi) is confined to soils with significant reserves of organic matter and rich vegetation. With respect to this interesting observation, basidiomycete fungi were found exclusively in the moss–lichen tussock tundra, which indicates the high specificity of this biotope.

### 3.5. CFU Number of Microfungi

The CFU number of microfungi in the topsoil horizons ranged from 0.53 to 13.9 × 10^3^ CFU/g soil (Table 2). In Entic Podzol soils, the average number of soil microfungi reached 9.92 × 10^3^ CFU/g. These values were close to the lower limit of number for the background tundra soils of the Kola Peninsula, which varied from 8 to 328 × 10^3^ CFU/g [36]. In the Entic Podzol (RY01), the largest number of microfungi (13.9 × 10^3^ CFU/g) was observed in the topsoil horizon, which then significantly (more than 50 times) (t = 2.5, p = 0.05) decreased in the subsoil horizons. This is primarily due to the high abundance of organic matter in the humus horizon. In Albic Podzol (RY07), the CFU number was lower than in Entic Podzol and decreased by more than 40 times from the topsoil horizon to the subsoil one. Such a regularity in the distribution of microfungi along the soil profile was previously noted by us for the background Podzol [32,33]. The smallest number of culturable microfungi was found in anthropogenically disturbed soils and in Histosols. The environmental conditions (pH, TOC content), disturbance of the natural composition of soils and change in vegetation type led to a change in the structure of the soil microorganism’s community. The number of microfungal CFU in cryogenic spot was the same in topsoil horizon in Albic Podzol. The proportion of psychrophilic fungi was 30–60% of the total number of isolated culturable fungi in the soil of Rybachy Peninsula.

### 3.6. Number of Bacteria

The number of copiotrophic bacteria in the topsoil horizons of the Rybachy Peninsula ranged from 41.8 × 10^3^ cells/g to 5.5 × 10^6^ cells/g, with oligotrophs ranging from 77.9 × 10^3^ cells/g to 12 × 10^6^ cells/g (Table 2), which indicates the predominance of the oligotrophic group, which is characteristic of soils in the northern regions [17,33]. The abundance of both groups of bacteria reaches the highest values in the topsoil horizons. Then, there is a decrease in number by an order of magnitude in the subsoil horizons. Most of the bacteria (50–70%) were psychrotolerant, which is confirmed by their ability to grow on nutrient media at low temperatures.

The number of culturable bacteria reached the highest value in the topsoil horizon in anthropogenically disturbed soil, amounting to 12 million cells/g and almost an order of magnitude higher than that in the Entic Podzol and Albic Podzol. The lowest number of bacteria was revealed in the cryogenic spot soil.

The number of bacteria in the topsoil horizons of other soil types ranged from 210 × 10^3^ to 1 × 10^6^ cells/g for the copiotrophic group and from 420 × 10^3^ to 3.3 × 10^6^ cells/g for the oligotrophic group of bacteria.

An increase in the number of culturable bacteria in the Folic Histosol (Technic) may indicate anthropogenically modified of the soil. A high number of bacteria in anthropogenically disturbed soils was noted in other studies of urban soils carried out both for the Murmansk region and for the middle zone [46]. Anthropogenically disturbed soils have a surface layer created as a human activity result, produced by mixing, filling, burying or contaminating material of urbanogenic origin [47]. Urban soils were characterized by a neutral or alkaline reaction and high organic matter content, which is favorable for the development of cultured bacteria [46,48,49].

Generally, the number of bacteria in the soil of Rybachy Peninsula corresponds to the data obtained for the soils of the Kola Peninsula [33]. At the same time, the data on the bacterial number obtained for the Rheic Histosol (513 × 10^3^ cells/g) were an order of magnitude lower than the data on the number of the copiotrophic bacteria characteristic of the upland and lowland peat soils of the southern taiga subzone of the European part of Russia and western Siberia [50]. Similar results were noted for Folic Histosol of the studied region with a bacterial number of 210–422 × 10^3^ cells/g and a predominance of oligotrophs, which receive fresh, nitrogen-poor organic matter. In the litters of typical Cryozem and Lithozem of Central Siberia, copiotrophs prevailed, their number exceeding 5 million cells/g [36].

### 3.7. Enzymatic Activity

The activity of soil enzymes is an integral indicator of the functional activity of soil microbiota. According to the scale of comparative assessment of enzymatic activity [51], the studied tundra soils can be classified as soils with high and very high biological activity, except for cryogenic spots. These values are consistent with the data obtained by us for tundra soils in the previous study [17].

**Figure 5:**
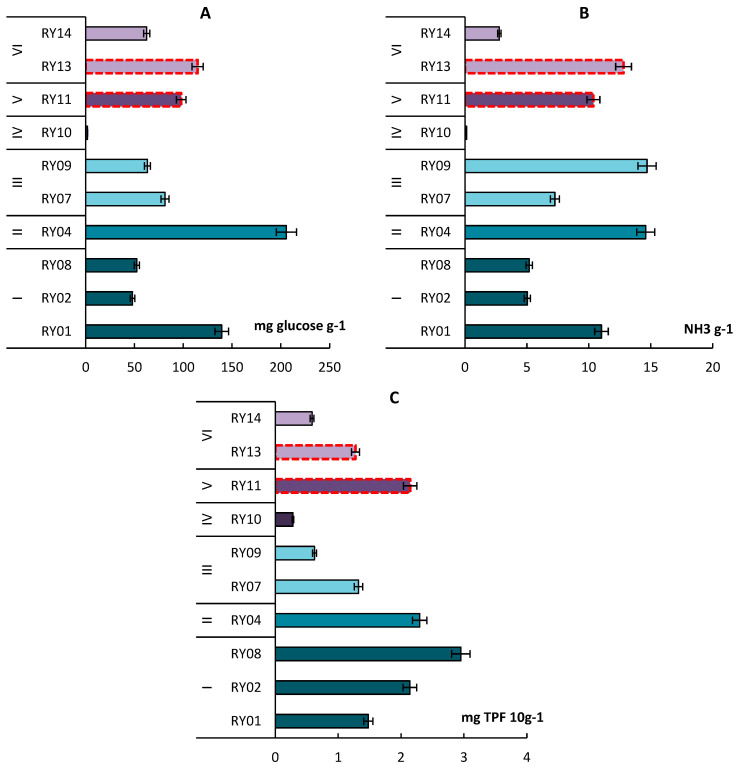
The activity of β-glucosidase (**A**), urease (**B**) and dehydrogenase (**C**) in topsoil of Skeletic Folic Leptic Entic Podzol (I), Rheic Leptic Rockic Histosol (II), Skeletic Folic Leptic Albic Podzol (III), Skeletic Leptic Entic Podzol—cryogenic spot (IV), Leptic Hemic Folic Histosol (Technic) (V), Leptic Hemic Folic Histosol (VI). The red dotted line indicates anthropogenically modified areas.

The maximum activity of hydrolytic enzymes noted in Rheic Histosol reached 205.7 mg glucose g-1 for β-glucosidase and 14.6 mg NH_3_ g^−1^ for urease (Figure 5A,B). The minimum enzymatic activity is typical for the cryogenic spot soil. The previous research showed that, in soil with many lignified roots and a high C/N ratio, a decrease in enzymatic activity and a slowdown in the decomposition of organic matter is possible [52]. This may be the reason for the lower activity of hydrolytic enzymes in the Folic Histosol soil in the crooked birch forest.

The dehydrogenase activity was maximum in the Entic Podzol in moss and lichen tundra at the top of the hill (RY08). Entic Podzol in shrub tundra and crooked birch forest (RY01, RY14) and Albic Podzol (RY09) were characterized by lower values of dehydrogenase activity (Figure 5C).

The activity of enzymes in anthropogenically modified soils (R11, R13) was at a high level and corresponded to or even exceeded the values obtained for some background tundra soils.

In accordance with the obtained values of enzyme activity, the studied tundra soils can be attributed to soils with high and very high biological activity (with the exception of cryogenic spots), which is confirmed by the scale for the comparative assessment of enzymatic activity [31] (Table S2). These values agree with the data obtained by us for tundra soils in our previous work [17].

The activity of β-glucosidase and urease in the studied soils is comparable or even higher than the values for the soils of more southern regions, while the activity of dehydrogenase is 2–5 times lower [53,54,55,56]. At the same time, when studying the enzymatic activity of soils in the Arctic (Bolshoy Solovetsky Island, Cape Beliy Nos, Vaigach Island, Novaya Zemlya Archipelago, Hooker Island of Franz Josef Land, Matveyev Island, Oranskie Islands), it was found that most soil samples had low urease activity [57]. The soils of the coastal and island territories of the White and Barents Seas (76% of the samples), in accordance with the scale of comparative assessment of the enzymatic activity of the soil, were also characterized by low urease activity [58].

It is believed that, in genetically different soils, as well as within the soil profile, there is a positive correlation between the activity of enzymes and the content of TOC [53,56,59]. In our research, the correlation analysis did not reveal any significant relationship between the activity of the enzymes and the content of organic carbon in genetically different soils. This was also shown in the study of sod–podzolic and gray forest soils [60].

The negative correlation (r = −0.71–0.83; t = 2.85–4.37; df = 8, p = 0.05) between the activity of hydrolytic enzymes and the pH value of the topsoil horizon was established. In more acidic soils, the activity of β-glucosidase and urease was higher. Authors of [60] also noted the correlation between the pH values of the water soil extract and the enzymatic activity of the soil, but the enzymatic activity was higher in soils with neutral pH level.

Analysis of the data did not show any significant effect of the structure of biomass of microorganisms on the activity of hydrolytic enzymes in different types of soils. A negative correlation with the biomass of microscopic fungi (r = −0.78; t = 4.05; df = 8, p = 0.05) was found for the dehydrogenase activity. High dehydrogenase activity was observed in areas with low fungal biomass. There is evidence that these enzymes are produced by soil bacteria of the genus *Pseudomonas*, the most common of which is *P. entomophila* [61,62]. However, in our study, no significant effect of prokaryotic biomass and number of bacteria on dehydrogenase activity was found.

The level of enzymatic activity of tundra soils cannot be explained only by the content of nutrients or microbiological parameters. The significant role of vegetation in the distribution of microbial and enzymatic activity of natural ecosystems soils was demonstrated, taking into account topographic and edaphic factors [63]. The activity of soil enzymes can also be influenced by soil texture. For example, dehydrogenase activity increases with a decrease in size of soil macroaggregates [64].

Within the profile of tundra soils in all areas, the maximum activity of enzymes was observed in the topsoil horizons. The soil enzymatic activity decreased down the profile, which positively correlates with the content of organic matter (r = 0.660–0.998) and negatively correlates with pH (r = −0.708–0.996). The level of significance for the correlations is 3.514–19.292 (df = 3, p = 0.05).

## 4. Conclusions

This study has shown that the tundra soils of the Rybachy Peninsula are generally characterized by a low biomass of microorganisms with a predominance of fungi, a high proportion of unicellular forms and nanoforms of prokaryotes, and the dominance of oligotrophic and psychrotolerant forms of bacteria. Despite the low abundance and biomass of microorganisms compared to more southern areas, tundra soils have a high enzymatic activity. Oligotrophy, low biomass, and the number of cultivated bacteria and fungi in the soils of the Rybachy Peninsula are adaptive characteristics of the microbiota of the Arctic regions. Under subarctic conditions, microorganisms can actively develop, populating substrates that are unsuitable for other life forms. Adaptation of the soil microbiome to the Subarctic conditions maintains a high enzymatic activity and generally determines the high biological activity of peat and dry peat soils.

A low biomass of fungi and bacteria, a low number of culturable fungi and a high number of bacteria, as well as a high proportion of actinomycetes characterize the anthropogenically modified tundra soils of the Rybachy Peninsula. Changes in natural soils because of anthropogenic impact and a change in vegetation types led to a change in the structure of the community of soil microorganisms. At the same time, enzyme activity remained at a high level.

The soils of the Rybachy Peninsula have a powerful enzyme pool due to the high adaptive potential of soil microorganisms to the extreme conditions of the Arctic, which allows them to perform their functions even under conditions of anthropogenic interference.

Similar comprehensive studies of the soil microbiome of the Rybachy peninsula have not been carried out. The enzymatic activity of soils in this region has not been assessed before. Therefore, the results obtained in this article complement the knowledge about the features of the Arctic soil microbiome and their functioning.

## Figures and Tables

**Table 1:** Characteristics of soil sampling sites on the Rybachy Peninsula.

Soil pit	Coordinates and Height above Sea Level	Type of Soil [20]	Horizons (Depth, cm)	Description of the Site
RY01	69°49′18.0″ N, 32°02′07.8″ E 35 m	Skeletic Folic Leptic Entic Podzol (Arenic)	O (0–20) Bs (20–40) BC (40–45)	Moss–lichen shrub tundra
RY02	69°49′18.3″ N 32°02′08.6″ E 34 m	Skeletic Folic Leptic Entic Podzol (Arenic)	O (0–15) Bs (15–45) BC (4–565)	Crooked birch forest shrub–forb
RY08	69°49′43.8″ N 32°02′37.0″ E 152 m	Skeletic Folic Leptic Entic Podzol (Arenic)	Oa (0–4) Bs (4–30) BC (30–50)	Moss and lichen tundra at the top of the hill
RY04	69°49′28.0″ N 32°02′12.0″ E 55 m	Dystric Rheic Leptic Rockic Histosol	H (0–35)	Eutrophic swamp
RY07	69°49′44.3″ N 32°02′31.5″ E 146 m	Skeletic Folic Leptic Albic Podzol (Arenic)	O (0–8) E (8–10) Bs (10–25) BC (25–50)	Shrub tundra, cryogenic crack
RY09	69°49′43.5″ N 32°02′47.6″ E 150 m	Skeletic Folic Leptic Albic Podzol (Arenic)	O (0–10) Oa (10–20) E (20–35) Bs (35–60)	Shrub tundra, cryogenic hillock
RY10	69°49′43.6″ N 32°02′42.0″ E 152 m	Skeletic Leptic Entic Podzol (Arenic)	Bs (0–7) BC (7–15)	Cryogenic spot, lichens and cereals
RY11	69°55′44.7″ N 31°58′08.5″ E 15 m	Dystric Leptic Hemic Folic Histosol (Technic)	Ou (0–10) BC (20–25)	Ruderal tall grass meadow, abandoned village, anthropogenic modified soils (chemical pollution and human impact)
RY13	69°53′37.4″ N 31°57′44.0″ E 6 m	Dystric Leptic Hemic Folic Histosol	O (0–10) BC (15–25)	Seaside forb meadow, anthropogenic modified soils (attempts at growing crops)
RY14	69°46′02.5″ N 32°05′56.8″ E 37 m	Dystric Leptic Hemic Folic Histosol	O (0–20)	Crooked birch forest

**Table 2:** Number of fungal and bacterial CFU (×10^3^, mean ± SD), measured using the plating method in the soils of the Rybachy Peninsula.

Soil Type	Soil Pit	Horizon	Fungi	Bacteria	
				Copiotrophic	Oligotrophic
Skeletic Folic Leptic Entic Podzol	RY01	O (0–10)	13.93 ± 3.5	814.3 ± 372.9	3310.3 ± 843.3
		O (10–20)	6.36 ± 2.5	993.4 ± 520.6	2254.6 ± 728.3
		Bs	1.77 ± 0.2	74.0 ± 30	342.0 ± 283.0
		BC	0.25 ± 0.03	8.6 ± 0.6	195.7 ± 26.0
	RY 08	Oa	5.9 ± 1.2	258.1 ± 113.9	672.5 ± 208.5
		Bs	2.87 ± 0.5	385.4 ± 174.7	781.5 ± 241.8
		BC	0.38 ± 0.03	74 ± 11.4	199.0 ± 84.8
Rheic Leptic Rockic Histosol	RY 04	H	0.53 ± 0.05	513 ± 146.0	736.0 ± 266.9
Skeletic Folic Leptic Albic Podzol	RY 07	litter	1.8 ± 0.03	513.9 ± 220.0	804.4 ± 344.0
		O	0.9 ± −0.005	159 ± 3.3	219.9 ± 48.4
		E	0.31 ± 0.04	5.5 ± 11.6	36.7 ± 49.1
		Bs	0.25 ± 0.02	10.2 ± 2.2	38.5 ± 17.5
		BC	0.04 ± 0.005	17.87 ± 5.1	17.3 ± 8.9
Skeletic Leptic Entic Podzol—cryogenic spot	RY 10	Bs	1.12 ± 0.4	41.8 ± 11.5	77.9 ± 22.7
		BC	0.5 ± 0.02	35.4 ± 11.9	161.5 ± 78.6
Leptic Hemic Folic Histosol (Technic) anthropogenic modified soils (chemical pollution and human impact)	RY 11	Ou	0.53 ± 0.10	5551.3 ± 627.7	12,059.6 ± 1029.5
		BC	0.05 ± 0.005	338.3 ± 95.3	376.8 ± 163.4
Leptic Hemic Folic Histosol anthropogenic modified soils (attempts at growing crops)	RY 13	O	0.53 ± 0.09	210 ± 98.8	422.7 ± 53.0
		BC	0.09 ± 0.003	46.65 ± 16.7	58.3 ± 28.3

## Data Availability

No new data were created or analyzed in this study. Data sharing is not applicable to this article.

## Supplementary Materials

The following supporting information can be downloaded at: https://www.mdpi.com/article/10.3390/microorganisms11051352/s1. Table S1. Structure of microbial biomass. Table S2. Scale for comparative assessment of enzymatic activity of soils [31].
