# Supplementary material for: Structure of Microbial Communities and Biological Activity in Tundra Soils of the Euro-Arctic Region (Rybachy Peninsula, Russia)
Source: Microorganisms. 2023 May 22;11(5):1352. doi: 10.3390/microorganisms11051352 (PMC10222164; doi:10.3390/microorganisms11051352)
Supplement: Supplementary file 1 [file microorganisms-11-01352-s001.zip › Table S1.pdf]

**Table S1.** Structure of microbial biomass.

| Soil pit | Fungi | | | | | | | | | | | Prokaryotes | | | | | | Total microbial biomass, mg/g | The proportion of fungi total microbial biomass, % |
|---|---|---|---|---|---|---|---|---|---|---|---|---|---|---|---|---|---|---|---|
| | Mycelium | | | Spores (diameter, µm) | | | | | Total spore biomass, mg/g | Total biomass of fungi, mg/g | The proportion of fungal mycelium in total fungal biomass, % | Actinomycete mycelium, m/g | | Unicelluler prokaryotes | | Total prokaryotes biomass, mg/g | The proportion actinomycete mycelium in total biomass, % | | |
| | | | | 2 | 3 | | 5 | | | | | | | | | | | | |
| | Biomass, mg/g | Lenght, m/g | Number, cells/g ×10^5 | Biomass, mg/g | Number, cells/g×10^4 | Biomass, mg/g | Number, cell/g×10^3 | Biomass, mg/g | | | | Biomass, mg/g | Length, m/g | Number, cells/g×10^8 | Biomass, µg/g | | | | |
| RY01 | 0.294± 0.049 | 232.88± 41.50 | 1.59± 0.30 | 0.053± 0.010 | 5.42± 0.97 | 0.063± 0.012 | 2.42± 0.41 | 0.013± 0.002 | 0.129± 0.024 | 0.423± 0.081 | 68.1 | 1.07± 0.20 | 27.31± 5.12 | 9.13± 1.67 | 40.31±7 .26 | 41.38± 12.05 | 2.6 | 0.539± 0.032 | 78.5 |
| RY02 | 0.159± 0.028 | 125.94± 22.09 | 1.44± 0.27 | 0.048± 0.009 | 4.99± 0.95 | 0.058± 0.011 | 3.16± 0.60 | 0.017± 0.003 | 0.123± 0.023 | 0.282± 0.054 | 56.4 | 0.57± 0.11 | 14.54± 2.65 | 6.12± 1.21 | 27.02±4 .86 | 27.59± 3.5 | 2.0 | 0.309± 0.005 | 91.3 |
| RY08 | 0.018± 0.003 | 14.26±0 2.08 | 1.11± 0.24 | 0.037± 0.007 | 9.83± 1.84 | 0.066± 0.012 | – | – | 0.103± 0.020 | 0.121± 0.023 | 14.9 | 0.20± 0.04 | 5.17± 0.95 | 2.25± 0.41 | 9.93±1. 79 | 10.13± 2.5 | 2.0 | 0.131± 0.060 | 92.4 |
| RY04 | 0.156± 0.027 | 123.57± 21.75 | 1.02± 0.20 | 0.034± 0.006 | 6.11± 0.95 | 0.041± 0.008 | – | – | 0.075± 0.014 | 0.231± 0.044 | 54.5 | 0.41± 0.08 | 10.45± 1.89 | 6.05± 1.10 | 26.71±4 .81 | 27.12± 3.8 | 1.5 | 0.258± 0.034 | 89.5 |
| RY07 | 0.143± 0.025 | 113.27± 19.66 | 1.26± 0.20 | 0.042± 0.008 | 10.73± 2.20 | 0.072± 0.014 | – | – | 0.114± 0.021 | 0.257± 0.050 | 55.6 | 0.29± 0.05 | 7.39± 1.40 | 12.56±2. 49 | 55.45±9 .98 | 55.74± 10.2 | 0.5 | 0.313± 0.040 | 82.1 |
| RY09 | 0.523± 0.098 | 413.67± 81.24 | 1.47± 0.28 | 0.049± 0.009 | 8.20± 1.59 | 0.055± 0.010 | 7.62± 1.49 | 0.041± 0.008 | 0.145± 0.027 | 0.669± 0.013 | 78.2 | 0.19± 0.04 | 5.07± 0.90 | 5.84± 1.12 | 25.78±4 .64 | 25.97± 4.3 | 0.7 | 0.695± 0.006 | 96.2 |
| RY10 | 0.337± 0.064 | 267.27± 50.43 | 1.17± 0.24 | 0.039± 0.007 | 7.06± 1.23 | 0.051± 0.010 | – | – | 0.090± 0.017 | 0.427± 0.083 | 14.4 | 2.49± 0.49 | 66.32± 12.71 | 2.72± 0.52 | 12.01±2 .16 | 14.50± 3.8 | 17.2 | 0.442± 0.032 | 96.6 |
| RY11 | 0.083± 0.015 | 65.67±0 12.57 | 0.96± 0.19 | 0.032± 0.005 | 5.36± 1.00 | 0.036± 0.007 | – | – | 0.068± 0.013 | 0.151± 0.028 | 55.0 | 1.11± 0.22 | 29.67± 5.14 | 2.13± 0.42 | 9.40±1. 69 | 10.51± 1.8 | 10.6 | 0.162± 0.005 | 93.2 |
| R13 | 0.108± 0.020 | 85.40±0 16.02 | 1.35± 0.28 | 0.045± 0.008 | 4.99± 0.95 | 0.058± 0.011 | – | – | 0.103± 0.020 | 0.211± 0.040 | 51.1 | 0.06± 0.011 | 1.47± 0.20 | 3.27± 0.64 | 14.44±2 .60 | 14.50± 2.3 | 0.4 | 0.226± 0.045 | 93.4 |

| | | | | | | | | | | | | | | | | | | |
|---|---|---|---|---|---|---|---|---|---|---|---|---|---|---|---|---|---|---|
| RY14 | 0.321± 0.062 | 254.19± 48.78 | 1.53± 0.29 | 0.051± 0.009 | 6.11± 0.95 | 0.041± 0.008 | 11.33± 2.03 | 0.061± 0.012 | 0.153± 0.028 | 0.474± 0.094 | 67.7 | 0.67± 0.013 | 17.91± 3.36 | 2.42± 0.47 | 10.68±1 .92 | 11.35± 0.8 | 5.9 | 0.485± 0.123 | 97.7 |

Note: "-" sign means "not found"
