# Supplementary material for: Structure of Microbial Communities and Biological Activity in Tundra Soils of the Euro-Arctic Region (Rybachy Peninsula, Russia)
Source: Microorganisms. 2023 May 22;11(5):1352. doi: 10.3390/microorganisms11051352 (PMC10222164; doi:10.3390/microorganisms11051352)
Supplement: Supplementary file 1 [file microorganisms-11-01352-s001.zip › Table S2.pdf]

Table S2. Scale for comparative assessment of enzymatic activity of soils [31]

Enzymatic activity	Dehydrogenase, mg TPF 10 g^{-1}	Β-glucosidase, mg glucose g^{-1}	Urease, mg NH_3 g^{-1}
Very low	<0.2	<5	<3
Low	0.2-0.5	5-15	3-10
Medium	0.5-1.0	15-50	10-30
High	1.0-1.5	50-150	30-100
Very high	>1.5	> 150	> 100
